# Coping with primary brain tumors together: a scoping review of dyadic psychosocial interventions

**DOI:** 10.1007/s00520-026-10676-0

**Published:** 2026-04-16

**Authors:** Chiara Acquati, Nenette A. Caceres, Karen Clark, Alejandro Fernandez, Jana Portnow, Lisa Feldman, Stephanie Yoon, Behnam Badie, Matthew Loscalzo, William Dale

**Affiliations:** 1 Eli Coleman Institute for Sexual & Gender Health, University of Minnesota Medical School, Minneapolis, MN 55455 USA; 2 Department of Family Medicine and Community Health, University of Minnesota Medical School, Minneapolis, MN 55455 USA; 3 Graduate College of Social Work, University of Houston, Houston, TX 77204 USA; 4 Department of Health Disparities Research, The University of Texas MD Anderson Cancer Center, Houston, TX 77204 USA; 5 Department of Supportive Care Medicine, City of Hope National Medical Center, 1500 E Duarte Road, Duarte, CA 91010 USA; 6 Department of Medical Oncology & Therapeutics Research, City of Hope National Medical Center, Duarte, CA 91010 USA; 7 Division of Neurosurgery, Department of Surgery, City of Hope National Medical Center, Duarte, CA 91010 USA; 8 Department of Radiation Oncology, City of Hope National Medical Center, Duarte, CA 91010 USA

**Keywords:** Glioma, Primary brain tumors, Dyadic interventions, Care-partners, Couples, Supportive care, Distress, Coping, Quality of life, Communication

## Abstract

**Purpose:**

Gliomas are associated with poor prognosis and place significant emotional, psychological, and practical burdens on patients and their care-partners. Dyadic interventions hold promise for mental health, coping, and quality of life. This scoping review synthesizes the current landscape of dyadic interventions in glioma and neuro-oncology care, examining intervention characteristics, theoretical foundations, and psychosocial outcomes, while identifying gaps to guide future research and clinical practice.

**Methods:**

A systematic search (2013–2024) of PubMed, EMBASE, Cochrane, CINAHL, and PsycINFO was conducted for English-language studies. Using the PICOS framework, we included studies involving individuals with brain tumors and their romantic/intimate partners. Eligible studies reported psychosocial, health-related, feasibility, acceptability, or efficacy outcomes for both members of the dyad. Studies were excluded if partners comprised less than 20% of the caregiver sample.

**Results:**

Eleven publications met the inclusion criteria. Interventions included yoga, meditation, psychoeducational and CBT models, dignity therapy, EMDR, and communication coaching. Programs ranged from in-person to online, and from single sessions to multi-week. Across studies, feasibility and acceptability were confirmed, with observed benefits in emotional distress, caregiver mastery, relational connection, and existential well-being. However, many were early-phase and methodologically heterogeneous, with inconsistent reporting of participants’ characteristics, outcome measures and evaluation of mechanisms of change.

**Conclusions:**

Although interest in the application of dyadic approaches to glioma care is increasing, the evidence base remains limited and fragmented. Advancing this field will require more rigorous, theory-driven interventions, including standardized outcome measures and perspectives from patients, partners, and providers to ensure relevance, feasibility, and clinical applicability.

## Introduction

Primary brain tumors (gliomas) are cancers that can range from slow-growing, low-grade tumors to highly aggressive forms like glioblastoma multiforme (GBM). Gliomas account for nearly 80% of malignant brain tumors [1]. Standard treatment typically includes a combination of surgery, radiation therapy, and chemotherapy, aiming at safeguarding cognitive abilities and quality of life [2]. Current treatment options for gliomas, particularly high-grade glioblastomas, remain limited in efficacy, providing only modest survival benefits, as the median survival for patients with GBM is approximately 12–20 months [3]. Post-treatment side effects, such as headaches, seizures, cognitive impairment, personality changes, and motor dysfunction, significantly impact patients' quality of life. Patients and their loved ones face profound physical and psychological challenges throughout the illness. Escalating care responsibilities and psychosocial distress can undermine the quality of life and relational stability of patients and their partners, limiting their capacity and resources for coping with these complex issues [4–6].

Recent evidence suggests that supportive, psychosocial, and rehabilitative interventions, such as physical therapy, psychoeducation, cognitive behavioral therapy (CBT), mindfulness-based stress reduction, and caregiver support education, can significantly enhance the quality of life for patients and caregivers (hereafter referred to as "dyads") [7, 8]. Several studies have investigated dyadic interventions to enhance psychological well-being, emotional regulation and relationship quality across a variety of cancer types [9, 10]. While feasibility and acceptability are often demonstrated in pilot studies, these programs largely remain limited in scope, underpowered, and heterogeneous in their theoretical underpinnings and selected outcome measures. The lack of guidance on optimal timing, delivery method, “dosage” (or intensity) of interventions makes it challenging to determine continued use, integration into clinical care pathways, and maintenance of effects over time [11–14]. These findings suggest a knowledge gap and an urgent need for targeted approaches to enhance the quality of life for dyads facing primary brain tumors.

Critical gaps also exist in terms of inclusivity and accessibility, with studies finding disparities in age, race, and access to specialized care leading to delayed treatment and worse survival [15–17]. Many interventions fail to account for cultural, socioeconomic, or linguistic diversity among dyads. The evolving needs of dyads across different disease stages, from diagnosis to recurrence or end-of-life care, are also rarely reflected in the design or delivery of interventions. Family support and caregiver-patient relationship dynamics are crucial in treatment adherence and clinical outcomes [15, 16, 18, 19]; however, caregiver outcomes are often underreported in studies. Psychosocial interventions targeting patients and caregivers have shown considerable promise due to their ability to reduce distress, enhance communication, and promote well-being in both patients and caregivers [4, 20–25]. In the context of primary brain tumors, video-based educational resources were found to reduce emotional and informational burden among caregivers [26]. Similarly, a nurse-led psychosocial intervention has been shown to improve satisfaction, emotional preparedness, and coping; which can translate to better adherence and quality-adjusted survival [26]. These findings underscore the importance of systematically examining the current literature about psychosocial interventions among glioma patients and their caregivers.

This scoping review synthesizes existing dyadic programs, with specific attention to interventions targeting symptom burden, psychosocial and relational outcomes among patients and care-partners. Additionally, this contribution further identifies gaps and future directions to guide the refinement and sustained implementation of dyadic programs in cancer care. The following research questions guided our review:

- What dyadic psychosocial interventions have been documented in the care of patients and care-partners facing primary brain tumors, and in what clinical contexts and populations have they been applied?
- What key features define dyadic interventions in this area, with respect to their format, delivery, outcomes, and theoretical or conceptual foundation?
- Which interventions have been shown to reduce psychological distress and symptom burden, and enhance communication and relationship quality in patients with primary brain tumors and their primary support person?

## Methods

### Design

**Fig. 1:**
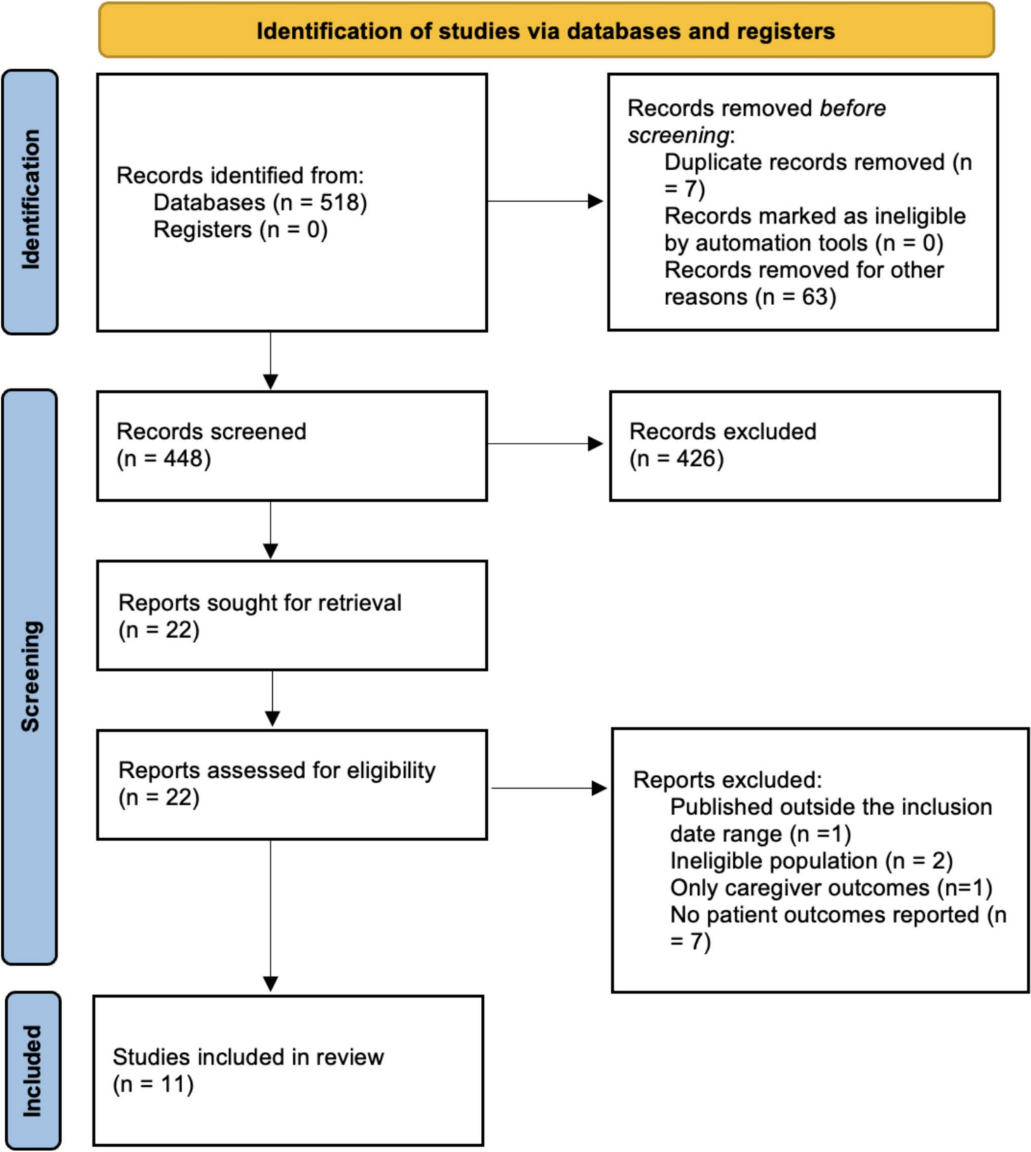
PRISMA 2020 flow diagram of database search and study selection process

The study selection (Table 1) and data extraction processes (Fig. 1) for this scoping review were guided by the Population, Intervention, Comparison, Outcome, and Study design (PICOS) criteria, which are outlined below:

**Table 1:** Overview of key concepts and search strategy keywords

Concept	Keywords
Dyad/Couple	*Dyad, dyads, dyadic, couple, couples, spouse, spouses, partner, partners, romantic partner, romantic partners, intimate partner, intimate partners, sexual partner, sexual partners, caregiver, caregivers, carer, carers, patient-caregiver dyad*
Glioma	*Glioma, brain cancer, brain tumor, brain tumour, primary brain tumours, astrocytoma, oligodendroglioma, glial tumour, glial tumor, glioblastoma multiforme, GBM, glioblastoma*
Communication	*Communication, disclosure, emotional disclosure, communication skills, holding back, avoidance, communication difficulties, communication barriers, protective buffering*
Distress	*Distress, depression, anxiety, stress, biopsychosocial distress, isolation, coping, guilt, regret, grief, patient reported outcomes, complicated grief, adjustment*
Quality of life	*Quality of Life, Health Related Qol*
Relationships	*Relationship functioning, relationship quality, relationship satisfaction, relationship closeness*
Psychosocial intervention	*Psychosocial intervention, psychosocial program, supportive care, supportive care intervention, behavior therapy, behavioral therapy, cognitive therapy, cognitive-behavioral therapy, couple therapy, couples therapy, couple program, couples program, psychoeducation, psycho-education, psychoeducational intervention, psycho-educational, psychotherapy, counseling, coping skills training, coping skills, neuropsychology, program, therapy, intervention*

### Population

Eligible studies focused on dyads consisting of care recipients and their primary support person. The care recipient was defined as an individual diagnosed with a primary brain tumor (i.e., glioma), and the support person was someone providing informal, unpaid care. Dyads were characterized by pre-existing personal, romantic, intimate, or sexual relationships, excluding cases involving a formal, trained healthcare provider (e.g., physician, trained peer mentor, employed paid caregiver). Records were excluded if partners comprised less than 20% of the primary support persons in the sample.

### Intervention

Inclusion criteria required interventions to offer psychoeducational, cognitive, behavioral, educational, and/or supportive/palliative care with a primary focus on psychological distress, symptom management, relationship functioning, and communication. Notably, couples therapy designed to enhance the overall relationship quality was excluded based on this criterion.

### Outcomes

Records were deemed eligible for inclusion if they presented any objective or self-report assessments of psychosocial, health, and/or feasibility, acceptability, and efficacy outcomes for both patients and partners/caregivers. The designation of outcomes as primary was contingent upon explicit labeling in the record or the presence of hypotheses specifying the outcome. These outcomes were categorized as follows: (1) *objective psychosocial or health measures*, involving data obtained through diagnostic interviews or chart reviews; (2) *self-report psychosocial or health measures*, encompassing data derived from self-reported questionnaires; (3) *rating scales provided by experts/providers or interventionists*; or (4) *feasibility/usability measures*, which could be either objective (e.g., retention rate) or self-reported (e.g., satisfaction survey).

### Study designs and comparators

Intervention studies inclusive of adults diagnosed with primary brain tumors/gliomas and their primary support person were considered. All trial designs, including single-arm trials, feasibility trials, and randomized controlled trials (RCTs), were eligible for inclusion. In RCTs, there was no restriction on the type of comparison condition, and waitlist control conditions were considered part of usual care for this review. All study analytic designs, including records reporting long-term follow-ups and secondary data analyses of trials, were eligible for inclusion. However, records exclusively discussing intervention development or study protocol without reporting the testing of the intervention with patient-caregiver dyads were excluded.

### Literature search

A comprehensive literature search was conducted to identify articles published in English between January 1, 2013, and June 30, 2024, utilizing the following databases: PubMed, EMBASE, Cochrane, CINAHL, and PsycINFO. The search was conducted by the librarian of the City of Hope Supportive Care Medicine Department (AL). The search was limited to studies published from 2013 onward because of the seminal work by Badr and Krebs [27], which provided a foundational understanding of dyadic approaches in oncology. Importantly, the team began working on this review in 2023, ten years after that publication. In the decade since that review, systematic reviews and meta-analyses have mostly focused on interventions targeting either patients or caregivers facing primary brain tumors, rather than dyadic models [28–30]. Because our aim was to provide a relationship-centered mapping of interventions, we intentionally began our search following the publication of the 2013 review. Seven broad concept categories (*dyads, primary brain tumor/glioma, communication, distress, quality of life, relationships, and psychological intervention*) were searched, and results were combined using the appropriate Boolean operators (AND, OR; See Table 1 for a detailed overview of the search strategy). For each domain, we used a broad range of keywords and related terms to capture the variability in terminology across the literature. Additionally, we planned to identify potentially eligible records by reviewing the reference lists of included articles and manually searching for published versions of results from protocol papers or abstracts that were not accessible during the initial screening.

### Article appraisal & selection

After eliminating duplicate articles, all the titles and abstracts of the studies were assessed by four coders (CA, WD, KC, and AF) to determine adherence to the eligibility criteria. Discrepancies were resolved by the senior, corresponding author (WD). CA, WD, and NAC read full-text articles to make a final determination of eligibility. Title, abstract, and full-text coding were conducted using an extraction spreadsheet adapted from Cochrane [31]. Coders were not blind to journals or study authors during screening, and reasons for article exclusion during the full-text review were recorded. The exclusion criteria encompassed: (1) *Lack of a fully dyadic intervention*, wherein the care recipient and a primary caregiver/support person were omitted; (2) *Studies categorized as intervention development, protocol articles, and/or those not reporting psychosocial, health-related, or feasibility/efficacy outcomes*; (3) *Absence of romantic* *partners *among the caregivers/primary support persons listed in the study; and (4) *Unavailability of the full text* manuscript for conference abstracts.

### Data extraction

Data were charted using the “Data collection form for intervention review – RCTs and non-RCTs” developed by The Cochrane Collaboration [31]. The review team tested the form in a sample of 5 studies to assess the form’s clarity and harmonize data extraction before full implementation. Charting was conducted independently by CA and NAC, and discrepancies were resolved through discussion with the senior author (WD). Extracted variables included general publication information (title, year, authors, journal, country, publication type, funding sources, and conflicts of interest, if reported), as well as detailed study characteristics such as design, participant demographics, intervention type, outcomes measured, and follow-up duration. Methodological data included study aims, design, timeline, and duration of participation. Population and setting details encompassed age, gender, recruitment methods, inclusion/exclusion criteria, illness severity, and co-morbidities. Data were collected on group allocation, descriptions of the intervention and comparator, duration of the intervention, delivery method, and provider qualifications. Outcomes were extracted as reported by the study authors, including time points, documentation sources, units of measurement, scales/measures used, results, and approaches to handling missing data. When applicable, risk of bias was assessed using the domains embedded within the Cochrane data extraction form [32]. This process involved evaluating key methodological features, including randomization procedures, blinding, and outcome reporting. The appraisal was used descriptively to contextualize the findings and  it did not influence study inclusion or data synthesis (Table 3).

## Results

Figure 1 displays the Preferred Reporting Items for Systematic Reviews and Meta-Analyses (PRISMA) flow diagram [33]. A total of 518 records were identified through database searches. Before screening, 70 records were removed (duplicate records, *n =* 7; removed for other reasons, *n =* 63), including conference proceedings (*n =* 8), abstract-only records (*n =* 10), non-English publications (*n =* 2), and other ineligible publication types/formats (*n =* 43). This left 448 records for title and abstract screening, of which 426 were excluded. Twenty-two full-text reports were assessed for eligibility; 11 were excluded for the following reasons: published outside the inclusion date range (*n =* 1), ineligible population (*n =* 2), caregiver-only outcomes (*n =* 1), and no patient outcomes reported (*n =* 7). Ultimately, 11 studies met the inclusion criteria and were included in the synthesis (Table 2).

**Table 2:** Summary of sample characteristics, intervention features, outcomes & measures, and key findings across included studies (*n =* 11)

Author (Year); Country)	N; Setting	Eligibility	Demographics (Pt/Cg)	Intervention (Duration; Provider)	Outcomes & measures	Key findings
Boele (2013); The Netherlands	65 dyads; Academic MC	WHO III–IV; life exp > 3 m; Cg ≥ 21 h/wk; ≥ 18; excl: lang/visual limits; unable CBT; < 3 m prog	Age (Int vs Ctrl): Pt 53 ± 11 vs 52 ± 9; Cg 51 ± 11 vs 51 ± 10; Sex: Pt 71% M (Int), 60% M (Ctrl); Cg 74% F (Int)	Structured psychoeducation + CBT for Cg (6 sessions/12 wks; psychologist)	Caregiver QOL, mastery, Mental health. Measures: SF-36; Pearlin Mastery Scale; GHQ; BN20; EORTC Brain Module	Psychoeducational + CBT intervention-maintained Cg QOL and significantly improved sense of control and MH vs standard care
Ketcher (2020); USA	10 dyads; NCI CCC	Primary brain CA; active tx; KPS ≥ 70; no severe cog impairment; Cg ≥ 18; consent	Age: Pt 56 ± 15; Cg 60 ± 11; Sex: 60% F both; Race: 95% NHW	Goal-based communication coaching (45–60 min; researcher; SW available)	Communication quality, relational insight, goal alignment. Pre/post structured communication & relationship questionnaires (custom)	Statistically significant improvements in communication effectiveness and alignment on future care goals; described as breakthrough in emotional expression
Korman (2021); Canada	17 pts (15 completed; 5 Cg); Cancer Ctr	Recurrent/progressed MBT; life exp 2w–1y; aware of prog; English; no cog impairment	Demographics not fully reported	Dignity Therapy (3 sessions/6 wks; trained MDT)	Communication, emotional expression, legacy. Semi-structured qualitative interviews	Dignity Therapy described as deeply meaningful; themes included improved communication, emotional catharsis, and legacy development
Milbury (2018); USA	5 dyads; NCI CCC	HGG; RT ≥ 4 wks; KPS ≥ 80; ≥ 18; English	Age: Pt 52; Cg 58; Sex: 80% F pts; Cg majority F; Race: majority Latino Cg	Pilot dyadic yoga during RT (12 sessions; certified instructor)	Cancer symptoms sleep, depressive symptoms, mental & physical QOL. MDASI; PSQI; CES-D; SF-36; BFI	Reduced fatigue, improved sleep quality, and alleviated physical and emotional symptoms in Pts; Cg reported improved emotional well-being and connection
Milbury (2019); USA	20 dyads; NCI CCC	Glioma I–IV; ≥ 20 RT fx; KPS ≥ 80; ≥ 18	Age: Pt 46; Cg 50; Sex: 50% F pts; 70% F Cg; Race: 85% NHW	Dyadic yoga during RT (12 sessions; 2 certified therapists)	Cancer-related symptoms, depressive symptoms, Mental Health, Cg fatigue. MDASI; CES-D; SF-36; BFI	Both Pts and Cg experienced improved mental health and reduced symptom burden; Cg reported decreased fatigue
Milbury (2020); USA	35 dyads; NCI CCC	Malignant glioma/brain mets; KPS ≥ 80; internet access; partner willing	Age: Pt 57; Cg 53; Sex: 54% M pts; 57% F Cg; Race: ~ 75% NHW	Online couple-based meditation (4 weekly 60-min sessions; counselor intern)	QOL, compassion, relationship well-being. MDASI-BT; CES-D; MAAS; Self-Compassion Scale; PAIR	Improved cognitive function, symptom management, and relational satisfaction in Pts; no significant improvements observed in Cg
Milbury (2023); USA	67 dyads; NCI CCC	Primary malignant glioma; RT ≥ 5 wks; KPS ≥ 80	Age: Pt 48; Cg 53; Sex: 63% M pts; 79% F Cg; Race: ~ 85% NHW	3-arm RCT: dyadic yoga vs Cg-only yoga vs UC (15 sessions/~ 6 wks; oncology-trained yoga therapists)	Feasibility; Cg QOL, depressive symptoms, caregiving esteem & burden. SF-36; BSI; CES-D; CRA; MAAS	Cg-only yoga produced greater reductions in burden and increased caregiving self-esteem vs dyadic or UC
Nordentoft (2022); Denmark	16 dyads; Rehab & Palliative Ctr	HGG; ≥ 18; Danish-speaking; residential rehab eligible	Median age: Pt 60; Cg 60; Sex: 53% M pts; 69% F Cg	Multimodal rehabilitative palliative program (4-day inpatient + follow-up; MDT)	Satisfaction, psychosocial needs, peer support, coping. EORTC-QLQ C30; distress eval; qualitative interviews	High satisfaction and perceived relevance; themes of social support, reassurance, and individualized information
Sharma (2021); USA	16 dyads; NCI CCC	Newly dx WHO III–IV; post-RT; ≥ 18; tablet-capable	Age: Pt 59; Sex: 50% F pts; 63% F Cg; Race: 88% NHW	3-arm feasibility RCT: Control vs PROQOL vs PROQOL + PC (6-mo longitudinal; clinicians + PC team)	Prognostic awareness alignment; QOL. PA Single-Item Tool; LASA-QOL	> 60% dyads completed 6-mo data; no reported burden. 68% Pt-Cg misalignment in prognostic awareness; low clinician-dyad discordance
Szpringer (2018); Poland	37 dyads; Oncology Ctr	Female GBM ≤ 3 m post-dx; outpatient; no prior psych tx	Median age: 63–66; 100% F	EMDR + standard care (10–12 sessions/~ 14 wks; certified therapist)	Anxiety, depression, anger, SOC. HADS-M; SOC-29	Significant reductions in anxiety, depression, and anger and increased SOC vs control; control group showed no improvement or worsening
Thakur (2019); India	80 dyads; Neurosurgery OPD	Post-op intracranial tumor; conscious at discharge; Cg present	Age (Exp vs Ctrl): 45 ± 14 vs 42 ± 13; mixed sex	Nurse-led counseling + educational pamphlet (single session; 4-wk follow-up)	Behavioral symptoms (number & severity) and Cg distress. NPI-Q; NPI-Q Distress Subscale	Significant reductions in behavioral symptoms and Cg distress vs control; control dyads worsened or remained impaired

Key acronyms used in this table: *BFI* Brief Fatigue Inventory, *BSI* Brief Symptom Inventory, *CBT* Cognitive Behavioral Therapy, *CCC* Cancer Care Center, *CES-D* Center for Epidemiologic Studies Depression Scale, *Cg* Caregiver, *EORTC-QLQ-C30* European Organisation for Research and Treatment of Cancer Quality of Life Questionnaire-Core 30, *GBM* Glioblastoma Multiforme, *HADS-M* Hospital Anxiety and Depression Scale – Modified, *KPS* Karnofsky Performance Status, *MAAS* Mindful Attention Awareness Scale, *MDASI* MD Anderson Symptom Inventory, *NCI CCC* National Cancer Institute Comprehensive Cancer Center, *NPI-Q* Neuropsychiatric Inventory-Questionnaire, *QOL* Quality of Life, *RCT* Randomized Controlled Trial, *SF-36* Short Form 36 Health Survey

### Sample, context and theoretical/conceptual underpinning of interventions

Selected contributions spanned the publication years 2013 to 2024. Interventions evaluated included dyadic yoga and meditation programs, psychoeducational models, dignity therapy, EMDR, and communication coaching. Across studies, the sample size varied widely, reflecting both feasibility/acceptability pilots and larger-scale intervention trials. The smallest cohorts were observed in early-stage or feasibility works (range: 5–16 dyads), whereas larger samples were recruited in controlled trials (range: 20–80 dyads) (Table 2). Only one study documented the sexual and gender identity of participants [34]. Dyads were the most common unit of allocation across treatment conditions, with caregivers most often being spouses or intimate partners. Some studies also included other caregiving relationships (Table 2). Across the four studies by Milbury and colleagues, patient age ranged from mid-forties to late-fifties. In the two yoga studies [35, 36], patients averaged 46–52 years, while caregivers tended to be older (approximately 50–58 years). In the couple-based meditation pilot RCT [34] patients averaged 57.5 years and caregivers averaged 53.2 years. In the 3-arm dyadic versus caregiver-only versus usual care RCT, mean ages were 48 years for patients and 53 years for caregivers. Nordentoft et al., Thakur et al., and Sharma et al. [29, 37, 38] reported a wider diagnostic age range. Szpringer et al. [39] included exclusively female participants, most of whom were in their 60s. Sex distribution varied across glioma studies, with some cohorts showing balanced representation (e.g., Milbury et al. [36]) and others demonstrating marked sex imbalance, including exclusively female samples [39] or predominantly male cohorts. In contrast, caregivers were overwhelmingly female across nearly all studies, often comprising 60–80% of caregiver samples, reinforcing well-documented gendered patterns of informal caregiving. Race and ethnicity reporting was variable but generally indicated predominantly Non-Hispanic White samples in U.S., Canadian, and European trials. Milbury et al. [40] enrolled approximately 85% Non-Hispanic White participants, Sharma et al. [37] reported 88% Non-Hispanic White, 6% Asian, and 6% Hispanic; and Ketcher et al. [41] reported 95% Non-Hispanic White and 5% Native American. Notably, Milbury et al. [34, 36] included a higher proportion of Hispanic participants among both patients and caregivers.

With respect to diagnosis, studies included individuals diagnosed with high-grade glioma (WHO Grade III–IV) or glioblastoma multiforme (GBM), reflecting the disease trajectory most associated with neurological decline, psychological burden, and caregiver distress. In contrast, Thakur et al. [38] included the most heterogeneous neuro-oncology sample (glioma, pituitary tumors, meningioma, schwannoma, and craniopharyngioma), with glioma representing 20% of the intervention and 33% of the control arm.

Interventions were delivered in specialized oncology settings (such as NCI-designated comprehensive cancer centers) [34–37], rehabilitation centers [38], and virtually [34, 37]. Recruitment across studies involved pragmatic clinic-based strategies, including identification through electronic medical records [35, 36, 40], direct approach during visits or consults [29, 41, 42], referrals from oncology providers and neuro-oncology teams [36, 37, 40], as well as recruitment at discharge [29].

### Intervention structure, delivery, and outcome measurement

Delivery modes included outpatient in-person formats (*n =* 3) [11, 38, 41], clinic-based consultation models (*n =* 2) [39, 42], outpatient in-person clinic-based delivery during radiotherapy (*n =* 2) [35, 36], hybrid in-person/videoconference delivery (*n =* 1) [34], residential rehabilitation (*n =* 1) [29], outpatient psychotherapy (*n =* 1) [39], and online delivery (*n =* 1) [34]. Session lengths ranged from 45–60 min [34, 35, 41] with intervention duration ranging from single sessions [38] to 6 months [37].

Interventions were delivered by a range of providers, including yoga therapists, oncologists/palliative care clinicians, social workers, psychologists/psychotherapists, multidisciplinary rehabilitation teams, and oncology nurses. Study designs included RCTs (*n =* 3) [11, 34, 40], feasibility/pilot RCTs (*n =* 3) [35–37], non-randomized controlled trials (*n =* 2) [38, 39], a prospective observational study (*n =* 1) [29], and qualitative/mixed-methods evaluations (*n =* 2) [41, 42]. Attrition was generally attributed to disease progression, particularly in advanced illness cohorts. Interventions varied in structure and delivery. Dyadic yoga programs were multi-session, therapist-led, and delivered during radiotherapy in outpatient oncology settings [35, 36], with one 3-arm RCT comparing dyadic versus individual yoga formats versus usual care [40]. Dignity therapy involved structured legacy-building conversations [42]. Dyadic goal-setting [41] constituted a brief, single facilitated discussion. Psychoeducation and CBT strategies were delivered in an RCT targeting caregiver mastery and quality of life [11]. A separate nurse-led counseling intervention targeted behavioral symptoms and caregiver distress [38].

Interventions were informed by cognitive-behavioral principles in Boele [11], mind–body approaches in Milbury [34–36, 40], and communication theory in Ketcher [41]. Dignity therapy was employed to facilitate legacy-building conversations, reinforcing a sense of identity and connection in advanced illness [42]. Symptoms and physical functioning were assessed using validated measures, including the MD Anderson Symptom Inventory (MDASI), Brief Fatigue Inventory (BFI), Karnofsky Performance Status (KPS), and Pittsburgh Sleep Quality Index (PSQI) [35, 36]. Overall physical and mental health were evaluated with the Medical Outcomes Study 36-Item Short Form Health Survey (SF-36) [11, 29]. Brain cancer-specific concerns were captured with the MDASI-BT and the EORTC Brain Cancer Module (BN20), administered alongside the EORTC QLQ-C30 [35, 40]. Subjective cognitive functioning was measured with the MOS Cognitive Functioning Scale [34], while behavioral and neuropsychiatric symptoms were evaluated using the Neuropsychiatric Inventory Questionnaire (NPI-Q) [11]. Emotional and psychological outcomes were assessed using The Center for Epidemiologic Studies Depression Scale (CES-D) [11], Brief Symptom Inventory (BSI) [11], and Hospital Anxiety and Depression Scale [29, 39]. Communication and existential concerns were evaluated through the Prognostic Awareness Single-Item Tool [37], while overall well-being was captured using the Linear Analog Self-Assessment Quality of Life Scale (LASA-QOL) [35, 36]. The Sense of Coherence Scale (SOC-29) measured the extent to which individuals perceive their circumstances as comprehensible, manageable, and meaningful [29]. Mindfulness-based coping and present-moment awareness were quantified using the Mindful Attention Awareness Scale (MAAS) [34], while compassion was assessed with the Self-Compassion Scale [34]. Intimacy was appraised only in one study with the Personal Assessment of Intimacy (PAIR) Inventory [34]. Caregiver competence and perceived control were assessed with the Caregiver Mastery Scale [11], while caregiving burden and positive carers’ experiences were assessed with the Caregiver Reaction Assessment (CRA) [11].

Risk of bias focused on several domains of RCT and non-RCT studies [32]. These included random sequence generation, which evaluates whether a truly random method was used to allocate participants; allocation concealment, which addresses whether group assignment was adequately hidden to prevent selection bias; and blinding of participants and personnel to assess the potential for performance bias. Incomplete outcome data was considered to determine how attrition or missing data were handled, while selective reporting examined whether all pre-specified outcomes were reported as planned. Lastly, other sources of bias, such as baseline imbalances or funding-related concerns, were also evaluated. Among the RCTs, concerns were most observed in the domains of blinding and incomplete outcome data, primarily due to small sample sizes and lack of blinded outcome assessment. Non-randomized and pilot studies generally showed a moderate to high risk of bias, particularly in areas related to confounding factors and adherence to the intervention (Table 3).

**Table 3:** Risk of bias summary for included studies (*n =* 11)

First author (Year)	Random sequence generation	Allocation concealment	Blinding of participants/personnel	Incomplete outcome data	Selective reporting	Other bias
Boele (2013)	Low	Low	High	Low	Low	Low
Ketcher (2020)	Unclear	Unclear	High	Low	Low	Low
Korman (2021)	Unclear	Unclear	NA	NA	NA	NA
Milbury (2018)	Unclear	Unclear	High	Low	Low	Low
Milbury (2019)	Low	Low	High	Unclear	Low	Low
Milbury (2020)	Low	Low	High	Low	Low	Low
Milbury (2023)	Low	Low	High	Low	Low	Low
Nordentoft (2022)	Unclear	Unclear	NA	Unclear	Unclear	Unclear
Sharma (2021)	Low	Unclear	NA	NA	NA	NA
Szpringer (2018)	Unclear	Unclear	High	Low	Unclear	High
Thakur (2019)	Low	Unclear	Unclear	Unclear	Unclear	Unclear

NA indicates “not applicable”. Risk-of-bias domains are adapted from the Cochrane RoB tool. See the Methods section for domain descriptions

### Effects of dyadic interventions on psychological, relational and quality of life outcomes

Across studies, the feasibility and acceptability of interventions were demonstrated, despite heterogeneous disease trajectories and varying intervention intensities. Yoga-based programs showed strong feasibility during radiotherapy, with high adherence and clinically meaningful improvements in symptom burden and sleep for patients and reductions in caregiver burden [35, 36]. Meditation-based interventions had similar results, with the couple-based program exhibiting high feasibility and satisfaction, completion rates, and preliminary efficacy for patients on symptom severity, compassion, and relational well-being [34]; although caregiver gains were modest. The comparison of dyadic versus caregiver-only yoga formats found that while dyadic delivery enhanced relational functioning, caregiver-only interventions yielded the strongest effects on caregiver quality of life [40].

Evidence on intervention efficacy in reducing distress was promising but limited by small sample sizes and methodological heterogeneity. Boele and colleagues [11] reported significant improvements in caregiver mastery and emotional quality of life following a structured support intervention. Milbury and colleagues [34] found an online couple-based meditation program feasible and acceptable, with improvements in relational satisfaction among patients [33]. Additionally, Szpringer and colleagues’ [39] pilot trial demonstrated reductions in anxiety and depression symptoms among glioblastoma patients following Eye Movement Desensitization and Reprocessing (EMDR), suggesting potential for trauma-informed approaches. Evidence from one of Milbury’s randomized trial indicated that dyadic yoga yielded greater relational benefits compared to individually delivered formats [40]. Although mechanisms of change were seldom formally evaluated, several studies implied that intervention effects may be driven by improvements in emotional regulation [39], shared coping strategies [35, 41], and enhanced mind–body awareness [34, 40]. No studies employed formal mediation or moderation analyses; however, Milbury and Ketcher identified demographic and relational factors -such as baseline relationship quality- as potential influences on intervention outcomes [35, 36, 41].

Communication-whether targeted explicitly or influenced indirectly-was highlighted across several studies. One single session intervention by Ketcher [41] facilitated more meaningful conversations, with participants reporting discussions on previously avoided topics [41]. Korman and colleagues [42] demonstrated that dignity therapy fostered emotional disclosure and dialogue through legacy-building conversations [42]. Although not explicitly communication-focused, Milbury and colleagues suggested that synchronized activities like yoga may support nonverbal communication and relational attunement [34–36, 40]. Additionally, the rehabilitative palliative care program by Nordentoft and collaborators [29] promoted dyadic coping and clarified caregiving roles in late-stage disease. Overall, findings suggest that even brief, structured interventions can enhance communication, particularly when they create space for shared reflection and emotional engagement. Interventions that directly involve both members of the dyad and address existential and relational themes appear promising for sustaining communication-related outcomes. Finally, it was observed that psychoeducation and CBT approaches preserved caregiver mastery and well-being [11], EMDR reduced anxiety, anger, and neuropsychiatric symptoms [39], and a nurse-led counseling program improved behavioral symptoms and decreased caregiver distress [38]. The residential rehabilitation model further illustrates feasibility and perceived benefit by strengthening shared coping and decreasing caregiver isolation [29].

## Discussion

This scoping review mapped the available evidence on dyadic interventions for individuals living with primary brain tumors and their care partners, with particular attention paid to intervention characteristics and outcomes. Eleven publications were included, representing a mix of randomized controlled trials, pilot studies, observational designs, and mixed-methods program evaluations. Across studies, dyadic interventions demonstrated overall feasibility and acceptability across delivery formats, with retention challenges largely associated with disease progression rather than intervention burden. These programs were implemented within specialized oncology clinics, rehabilitation centers, virtually, and home-based settings. Samples were predominantly middle-aged and racially homogenous, highlighting persistent gaps in inclusion and representativeness: participants were overwhelmingly Non-Hispanic White, except for studies conducted outside of the US or in settings, such as Houston, TX, characterized by diversity in racial and ethnic backgrounds. Diagnostic heterogeneity was minimal, with most trials focusing on high-grade glioma or glioblastoma multiforme (GBM). These findings underscore the need for more inclusive recruitment strategies and improved harmonization in the reporting of race, ethnicity, sex, gender and sexual identity/orientation variables.

Interventions varied substantially in structure and theoretical frameworks. Program duration ranged from single-session conversations to 12-week protocols. While some studies articulated explicit conceptual frameworks, others relied on implicit principles without formally stating the theories or models that informed intervention development, thereby limiting cross-study comparability and the understanding of underlying mechanisms.

Reductions in anxiety and depressive symptoms were reported in trials involving EMDR [39], CBT [11], meditation [34], and dyadic yoga [34, 36, 40], while dignity therapy offered existential relief and legacy-related benefits [42]. Interventions that explicitly targeted the dyadic relationship, such as couple-based meditation [34], dignity therapy [42], and structured goal-setting conversations [41], were associated with enhanced intimacy, emotional attunement, and collaborative coping. Improvements in quality of life were most evident in mindfulness and yoga-based approaches, whereas gains in caregiver mastery and perceived competence emerged primarily in CBT and psychoeducational interventions. Notably, digital delivery proved feasible and potentially scalable. Dose requirements varied, with multi-session formats common in mindfulness and yoga protocols, while dignity therapy and structured communication produced benefits with minimal session contact.

Results from this scoping review align closely with, and extend, the broader literature on non-pharmacological supportive care for individuals with primary brain tumors, while also refining conclusions drawn from the dyadic oncology literature generally. Consistent with O’Doherty et al. [30], psychosocial and complementary interventions can be feasibly implemented in neuro-oncology settings and may yield meaningful benefits across emotional, symptom management, and quality-of-life domains. Across both bodies of work, however, substantial heterogeneity in intervention content, delivery, and outcome measurement constrains definitive conclusions and emphasizes the need for greater conceptual and methodological standardization. This review also converges with conclusions drawn from the wider dyadic oncology literature [24, 25, 43, 44], which demonstrated that effectiveness varies according to outcome domain and intervention focus. For instance, while reductions in anxiety and depression were reported [43], Sun et al. [24] indicated that cancer-related distress of patients improved when interventions included skills training, communication, support, and in longer programs [24, 25], while Li et al. [21] reported inconsistent findings for anxiety, depression and distress in breast cancer. In contrast, caregiver-related outcomes and relational processes emerged as particularly responsive targets, reinforcing the premise that dyadic interventions may be well positioned to strengthen interpersonal functioning [24, 25].

The present study holds important implications for key stakeholders. For patients, this work supports the availability of interventions that attend to psychological and relational well-being, rather than focusing solely on medical treatment, illness progression, and/or symptom management. For partners, the synthesis highlights interventions tailored to their distinct and often underrecognized needs. For clinicians and healthcare providers, the reviewed contributions offer both practical models and a rationale for incorporating dyadic interventions into supportive care. At the same time, these findings emphasize the need for approaches that are theory-informed, inclusive, measurable, and scalable.

In summary, dyadic interventions appear to offer meaningful support for individuals coping with primary brain tumors and their care partners. To optimize their impact, future research must address gaps in theoretical frameworks informing these programs, methodological rigor, reporting of sample composition and outcome measurement. Furthermore, community-engaged approaches are essential to ensure interventions reflect the experience of dyads coping with primary brain tumors. Ongoing partnerships with patients, caregivers, clinicians, and advocacy organizations will be critical to shaping the content, delivery, and sustainability of these programs. Such collaboration not only improves relevance and responsiveness but also strengthens the likelihood that dyadic interventions will be integrated into real-world care delivery models.

### Limitations

Several limitations should be acknowledged. Despite a comprehensive search strategy across five major databases, relevant studies may not have been captured. Specifically, we excluded non-English-language articles and "in progress" or unpublished studies, which may have introduced language bias and publication bias. Though intentional to capture recent evidence, the date restriction (2013–2024) may have excluded earlier foundational work or more recent adaptations not yet indexed in selected databases. Second, although efforts were made to apply consistent inclusion criteria, the heterogeneity in terminology across studies (e.g., "caregiver," "partner," "dyad," "couple") may have affected study retrieval and comparability. Additionally, variations in outcome measures and inconsistent reporting across studies posed challenges for synthesis and interpretation of findings, limiting our ability to draw definitive conclusions about intervention effectiveness. Third, while the risk of bias was assessed using the Cochrane tool, the nature of included evidence meant that many findings were preliminary or underpowered, limiting generalizability. The heterogeneity in study designs, sample sizes, and populations reduces the applicability of findings across diverse neuro-oncology populations and care settings. Variations in outcome measures and inconsistent reporting across studies also posed challenges for interpretation. As with most scoping reviews, no meta-analysis or formal assessment of intervention effects was conducted. Therefore, we cannot quantify the magnitude of benefits or provide comparative effectiveness data. These limitations suggest that current evidence represents an early stage of dyadic intervention research in neuro-oncology. Conclusions should be interpreted as promising but preliminary, and findings should inform future research priorities rather than serve as definitive clinical guidance.

Given the prevalence of neurocognitive impairment among individuals with primary brain tumors (particularly those with GBM), future research should examine how cognitive status influences intervention engagement and benefit. Such work is essential for identifying which patients and dyads are most likely to benefit from dyadic approaches and for informing the development of adaptive or tiered intervention models. Notably, dyadic interventions may not be appropriate for individuals with limited or absent informal support networks, or in situations where caregiver preferences and patient priorities are misaligned. Future research should therefore examine alternative models of support for these populations, including individual-level psychosocial interventions, peer-support programs, structured caregiver coaching delivered independently of the patient, and integration of professional navigators or palliative care clinicians when informal support is insufficient. Screening for caregiver availability, relational strain, or decisional discordance at key transition points in neuro-oncology care may help tailor intervention allocation and prevent unintended burden. Additionally, hybrid or stepped-care approaches that allow patients and caregivers to participate jointly or separately based on relational readiness may enhance inclusivity and clinical feasibility. Developing adaptable, tiered models of psychosocial care that account for variability in dyadic functioning, preferences, and expressed needs, represents an important advancement for the field. 

## Conclusion

This scoping review identifies a limited, but nonetheless promising body of dyadic intervention research. While studies show potential benefits for both patients and caregivers, and nearly half employed randomized controlled designs, many were early phase, demographically homogeneous, with limited articulation of theoretical frameworks, and variations in measures and reporting of outcomes. Future work must prioritize rigorous, scalable models that address psychosocial and relational outcomes over time, reflecting the complex trajectory of neuro-oncology care.

## Data Availability

No datasets were generated or analysed during the current study.
